# *GATA4* transgenic mice as an *in vivo* model of congenital heart disease

**DOI:** 10.3892/ijmm.2015.2178

**Published:** 2015-04-09

**Authors:** HUA HAN, YU CHEN, GANG LIU, ZENGQIANG HAN, ZHOU ZHAO, YIN TANG

**Affiliations:** Department of Cardiovascular Surgery, Peking University People’s Hospital, Beijing 100044, P.R. China

**Keywords:** congenital heart disease, *GATA4*, atrial septal defect, transgenic mouse

## Abstract

Our previous study indicated that 8 patients from a family with a history of congenital heart disease had simple atrial septal defect (ASD) and carried the same mutation at codon 310 in the *GATA4* gene. In the present study, to identify the functional defects caused by this mutation in an *in vivo* model, the transgene DNA constructs were microinjected into mice to generate a transgenic mouse model. The mice were genotyped using PCR and DNA sequencing. Protein expression was measured by western blot analysis. qPCR was used to determine the copy number of the transgenes. The heart tissue was fixed and sectioned by conventional procedures. The Vevo 2000 system was used to perform echocardiography on the mice. The expression of *GATA4* target genes was measured using the real-time PCR system. The incidence of ASD in the heterozygous transgenic mice was found to be greater than that in the wild-type control mice (P<0.05). In addition, the expression of α-myosin heavy chain (*α-MHC*) in the heart tissues from the homozygous mice was lower than that in the heart tissues from their wild-type littermates (P<0.05). In conclusion, these results suggest that the introduction of *GATA4* M310V negatively affects the normal expression of *α-MHC*. In accordance with previous findings on *GATA4* mutation screening and *in vitro* experiments, this study confirms that *GATA4* M310V mutation may lead to the development of the congenital heart defect, ASD.

## Introduction

Congenital heart disease (CHD) is a multifactorial disease; genetic and environmental factors play a key role in the occurrence of this disease. It is considered that there are over 1,700 genes that are required for heart development. The genetically modified mouse is a good anatomical model for common cardiac malformations which is powerful experimental tool for understanding human CHD (1). The *GATA4* gene is essential to normal heart development. *GATA4* deficiency is embryonically lethal in mice, as this leads to severe ventricular developmental defects, resulting in cardiac malformation (2). *GATA4* also regulates the expression of cardiac structural genes, including α-myosin heavy chain (*α-MHC*), cardiac troponin C (*cTNC*), atrial natriuretic factor (*ANF*), brain natriuretic peptide (*BNF*), A1 adenosine receptor (*ADORA1*), muscarinic acetylcholine receptor M2 (*CHRM2*, cholinergic receptor, muscarinic 2), and angiotensin II 1A receptors (*AngII1a*) (3,4). When GATA4 zinc finger structures and other cardiac-specific transcription factors [NK2 homeobox 5 (NKX2.5), T-box transcription factor (TBX5) and myocyte-specific enhancer factor 2 (MEF2C)] interact to form complexes, these complexes play a role in transcriptional regulation and regulate heart development (5–8).

The *GATA4* gene is an important factor in the cardiac gene network and mutations in *GATA4* have been confirmed to be associated with the occurrence of atrial septal defect (ASD). *GATA4* has been used to distinguish patients with familial CHD from those with sporadic CHD (9–11). *GATA4* transgenic mice have been used to as a model to study the pathogenesis of human CHD. *GATA4* knockout is lethal to mouse embryos, which suffer severe cardiac malformation (2). Double heterozygous *GATA4-TBX5* mice exhibit atrioventricular septal defects (12), and double heterozygous *GATA4-GATA5* mice present with a phenotype of stenosis in the aorta and pulmonary artery (13). Heterozygous *GATA4* mutation may result in ASD phenotypes, ventricular septal defect, endocardial cushion defect, right ventricular dysplasia and cardiomyopathy (14). *GATA4* G295S transgenic mice generated by Misra *et al* presented with a normal abdominal cardiac morphology and cyclization pattern, but a weak ventricular myocardium and a single ventricle in homozygous mice, which died at the gestational age of 11.5 days; however, the heterozygous line propagated and one subset carried the phenotypes of semilunar valve stenosis and ASD (15). This indicated that the complete lack of *GATA4* gene activity has a severe impact on cardiac development in mice, which cannot survive or reproduce. The homozygous animal model limits the functional study of *GATA4* and to a certain extent cannot represent human disease. However, heterozygous transgenic mice have phenotypes that resemble, reasonably closely, human CHD phenotypes, which are caused by genetic mutations.

Our group discovered a representative family with simple ASD in 2007 (9). Since then, thorough individual examinations were performed (e.g., collection of medical history, physical examination and echocardiography) upon a total of 31 family members from 1st- to 3rd-level relatives. A total of 8 patients, including the probands, were diagnosed with simple ASD. Subsequently, an in-depth study on the mechanisms of *GATA4* M310V mutation associated with familial ASD by our group revealed that *GATA4* M310V mutation affects *α-MHC* promoter activity in *in vitro* experiments (16).

In the present study, in order to identify the site of *GATA4* mutation capable of producing functional defects in an *in vivo* model, a *GATA4* M310V same-point mutation was generated in transgenic mice. The mice were bred and screened, producing a stable transgenic mouse line. We found that the incidence of ASD in the heterozygous transgenic mice was higher than that in their wild-type control littermates (P<0.05). In addition, the peak pulmonary artery pressure (PPAP) and speed in the heterozygous mice were higher compared with their wild-type control littermates (P<0.05). The expression levels of *GATA4* downstream target genes (*α-MHC*) in the homozygous mice were lower than those in their wild-type control littermates (P<0.05). To summarize, *GATA4* M310V mutations were associated with a higher incidence of ASD-like cardiac malformation in heterozygous transgenic mice compared with the wild-type controls. Due to the changes in the expression of downstream target genes in the homozygous transgenic mice, it was inferred that the *GATA4* M310V transgenic mouse model may be used to simulate ASD reasonably well. The effects of mutant *GATA4* M310V were found to involve downstream genetic changes that may result in the development of CHD.

## Materials and methods

### Construction and breeding of GATA4 M310V transgenic mice

According to the human GATA4 encoding sequence, we performed gene synthesis, followed by site-specific mutagenesis and construct amplification in the pcDNA3.1-N-Myc-GATA4 (A928G) vector. The DNA constructs were then microinjected into mouse embryos to generate *GATA4* M310V transgenic C57BL/6 mice. Each F0 transgene-positive transgenic mouse represented an individual transgenic line and was housed in a cage with wild-type mice (each cage contained 2 females and 1 male). All transgenic mice were genotyped by polymerase chain reaction (PCR) to screen for positive transgenic animals. The PCR products were verified by DNA sequencing. The animals were bred using the aforementioned method until the 4th generation. All animal experiments were carried out according to the approval of the Animal Studies Ethics Committee of the Peking University People’s Hospital, Beijing, China.

### Evaluation of the genetic stability of the exogenous genes of GATA4 M310V transgenic mice

Fluorescence-based quantitative PCR (qPCR) was used to determine the copy number of transgenes in the mouse model and to further screen a stably inherited transgenic line in this study. The stably inherited transgenic mouse line was selected by qPCR based on the following criteria: the genomic DNA of wild-type mice was used as a control, the genomic DNA of transgenic mice was used as a test sample, and *GATA4* and *GCG* were used as target fragments. Following confirmation of the sequence, the *GATA4* M310V plasmid DNA and GCG plasmid DNA were 10-fold serially diluted with sterile distilled water to produce 10^9^, 10^8^, 10^7^, 10^6^, 10^5^, 10^4^ and 10^3^ copy/*μ*l standards. qPCR was used to amplify 2 sets of standards (the templates for *GATA4* and *GCG* genes) and to generate a standard curve for amplification efficiency. The 2^−ΔΔCt^ method (17) was used to measure the exogenous gene copy number of the transgenic mice. The formula was as follows: 2^−ΔΔCt^ = 2^−[(Ct GATA4 - Ct GCG)] in transgenic samples - [(Ct GATA4 - Ct GCG)] in wild-type samples^.

### Assessment of GATA4 protein expression

A total of 3 (4-week-old) F3 transgene-positive transgenic mice and their 3 wild-type littermates were selected. The protein expression of GATA4 in the heart tissue was measured by western blot analysis using glyceraldehyde 3-phosphate dehydrogenase (GAPDH) as an internal reference. The rabbit polyclonal antibody to GATA4 (ab84593; Abcom, Cambridge, UK) which reacts with the human one was selected at the concentration of 1 *μ*g/ml to assess GATA4 protein expression.

### Comparative study of cardiac morphology and functions between transgenic and wild-type mice

The dead bodies of newborn heterozygous transgenic mice were collected [approximately one tenth of the heterozygous transgenic mice died shortly after birth (0–5 days after birth)]. Homozygous newborn mice and their wild-type littermates were euthanized by carbon dioxide inhalation. The animals were dissected by cutting open the chest to expose and remove the heart by cutting the root of the major arteries. The dissected hearts were rinsed in 0.01 mol/l phosphate buffer to remove the blood and were immediately placed in freshly prepared 4% neural buffered formalin. The geart tissue was then fixed for 18 h, followed by conventional dehydration, paraffin immersion and embedding procedures. The paraffin-embedded tissue was serially sectioned into 4-*μ*m-thick slices and stained in hematoxylin and eosin (H&E) solution. The 8-week-old heterozygous transgenic mice and their wild-type littermates were selected and anesthetized with sodium pentobarbital (50 mg/kg body weight). The cardiac index was calculated and following imaging of the mice, their heart rates were carefully maintained at approximately 490 beats/min. An ultra-high resolution and real-time ultrasonic molecular imaging system (Vevo 2100; VisualSonics, Toronto, ON, Canada) was used to perform M-mode echocardiography, two-dimensional echocardiography and color Doppler echocardiography on the mice. Images of apical four-chamber view (A4C), parasternal long axis view of the left ventricle, and parasternal short axis view of the left ventricle (at the aortic valve level) were then collected. The basic data measurement and calculation of the heart and parameters of cardiac function were obtained using conventional methods. The experimental procedures were performed under double-blind conditions with regard to genotype. The Chi-square test and Fisher’s exact test were used to determine the difference in the incidence of ASD. The Student’s t-test was used to compare the independent sample results of echocardiography. P<0.05 represents a statistically significant difference.

### Assessment of GATA4 downstream target gene expression

Total RNA from the heart tissues of the homozygous transgenic mice and their wild-type littermates (control mice) was extracted using TRIzol^®^ reagent (Life Technologies, Grand Island, NY, USA). Two micrograms of RNA were immediately reverse-transcribed into cDNA using the Promega Reverse Transcription kit (Promega, Madison, WI, USA). The expression levels of the *α-MHC*, *cTNC*, *GATA6*, *NKX2.5* and *TBX5* genes were measured using the Applied Biosystems StepOnePlus™ real-time PCR system using the KAPA SYBR^®^ FAST qPCR kit Master Mix (2X) Universal (Kapa Biosystems, Worburn, MA, USA). The primer sequences are listed in Table I. The relative average gene expression in the cardiac tissues of the transgenic mice and their wild-type littermates was calculated using *GAPDH* as an internal reference and the 2^−ΔΔCt^ formula. Each sample was evaluated in triplicate on a qPCR plate. SPSS 13.0 statistical software (SPSS, Inc., Chicago, IL, USA) was used to perform non-parametric tests to screen 2 independent samples. P-values <0.05 were considered indicative of statistically significant differences.

## Results

### Establishment and breeding of GATA4 M310V transgenic mice

**Figure 1:**
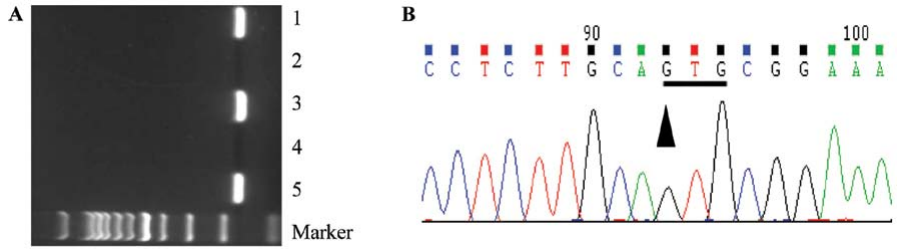
(A) Agarose gel image illustrating the genotyping of the transgenic mouse model. The size of the PCR product was 168 bp. The arrow indicates the 200 bp size of the molecular marker. Lanes 1,3,5 represent the genomic DNA products of 3 positive transgenic mice after genotyping and PCR amplification. Lane 2 represents the corresponding region in the wild-type littermate that had no amplified PCR fragment. Lane 4 represents the negative result of water. This animal was used as a negative control in this experiment. (B) Direct DNA sequencing to confirm the presence of mutation. The arrow indicates the corresponding methionine to valine amino acid substitution of *GATA4* M310V point mutation.

All transgenic mice were genotyped using PCR to screen for positive transgenic animals (Fig. 1A). Genomic DNA was verified by sequencing (Fig. 1B). The animals were bred using the aforementioned method (as described in the Materials and methods) to the 4th generation. A stably inherited transgenic line was screened. The positive heterozygous transgenic mice were inbred to generate homozygous transgenic mice.

### Evaluation of genetic stability of exogenous genes in GATA4 M310V transgenic mice

**Figure 2:**
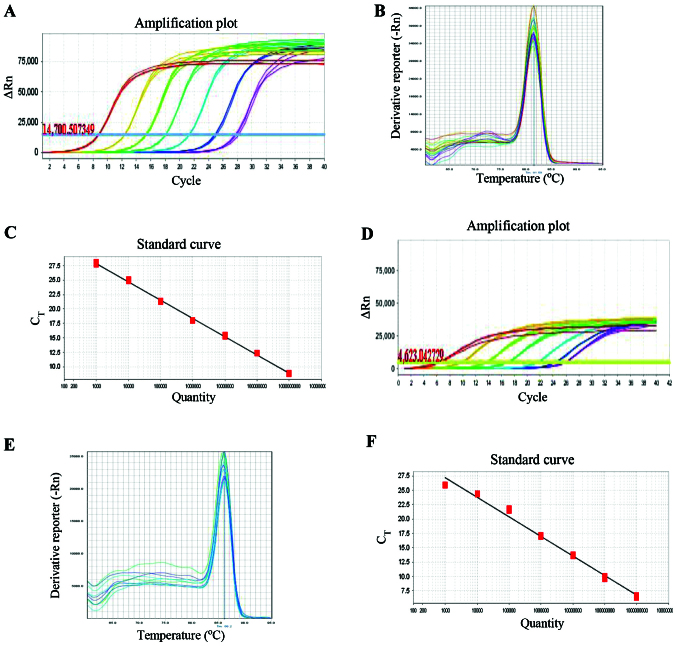
(A and D) Amplification curves; (C and F) standard curves; (B and E) melting curves of 10^2^, 10^3^, 10^4^, 10^5^, 10^6^, 10^7^ and 10^8^ copies of the mutant *GATA4* M310V plasmid and single copy *GCG* gene.

The *GATA4* M310V application and melting curves are illustrated in Fig. 2C and D, and the *GATA4* M310V standard curve is illustrated in Fig. 2F. No sign peaks were found in the melting curve and no primer dimers or other non-specific amplification were observed. The data obtained by PCR amplification were therefore considered reliable. The correlation coefficient of the *GCG* standard curve coefficient was R^2^=0.999 with 101% amplification efficiency, and the correlation coefficient of the *GATA4* standard curve was R^2^=0.988, with 97% amplification efficiency. The 2^−ΔΔCt^ method of qPCR revealed that the amplification efficiency between the reference and target genes was very close, ranging from 90–110%.

### Protein expression of GATA4

**Figure 3:**
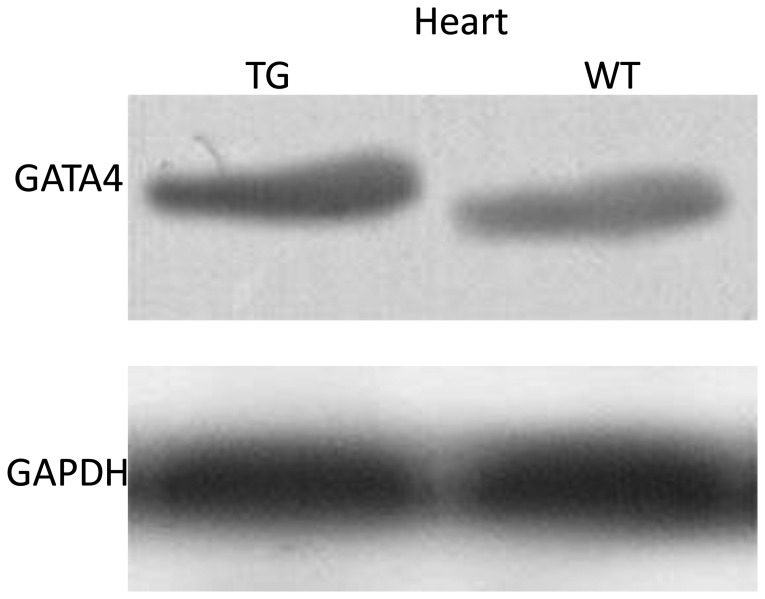
Representative result of western blot analysis. GATA4 protein expression in wild-type and F3 transgene-positive transgenic mouse hearts. In transgenic mouse hearts, the protein expression of GATA4 was increased (P<0.05). TG, transgenic; WT, wild-type.

To assess the changes in GATA4 protein expression in the hearts of F3 transgene-positive transgenic mice, western blot analysis was used to analyze the protein expression levels of GATA4. The results revealed that the protein expression level of GATA4 was higher in the hearts of the transgenic mice than in those of the wild-type mice (P<0.05; Fig. 3).

### Cardiac phenotype of heterozygous transgenic mice Observation of cardiac structure in newborn mice

**Figure 4:**
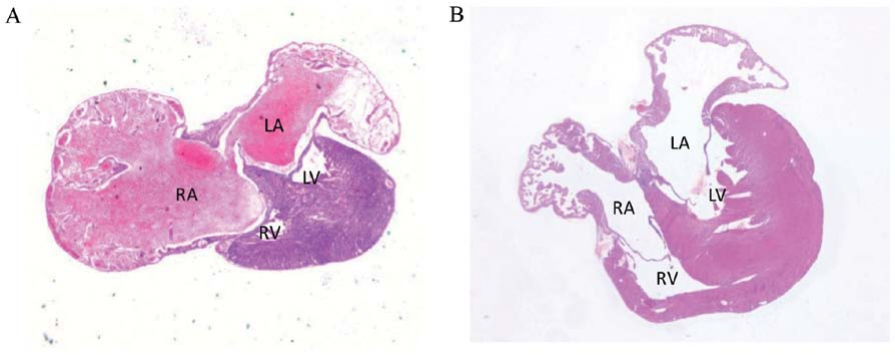
Hematoxylin and eosin (H&E) staining of coronal heart sections in the newborn mice. (A) Representative coronal heart autopsy section of neonatal heterozygous transgenic mouse. It shows an incomplete atrial septum with connective blood cells between the left and the right atria, but no obvious morphological abnormality of the myocardial cells. (B) Representative coronal heart sections of the wild-type littermates. No obvious abnormalities in cardiac tissue structure and myocardial cell morphology were observed. (A) Newborn heart of *GATA4* M310V transgenic mouse. (B) Newborn heart of wild-type control mouse. LA, left atrium; RA, right atrium; LV, left ventricle; RV, right ventricle.

Approximately one tenth of the heterozygous transgenic mice died shortly after birth (0–5 days after birth). To observe the cardiac structural defects of the *GATA4* M310V heterozygous transgenic mice, the dead newborn pups were collected and their hearts were dissected and subjected to conventional histological analysis. H&E staining of the serial heart sections revealed that, among the 15 newborn heterozygous mice, 9 mice (60%) had the ASD phenotype in their hearts (Fig. 4A) and 6 mice (40%) had no obvious structural defect. A total of 20 newborn wild-type littermates were selected as the controls and only 1 mouse (5%) had the ASD phenotype in its heart. The remaining mice had no obvious structural defects (Fig. 4B). The details of the transgenic and wild-type mice regarding cardiac structure are presented in Table II, suggesting that *GATA4* M310V produces the phenotype of ASD in the heart.

### Echocardiography for the diagnosis and assessment of cardiac structure and function in the mice

**Figure 5:**
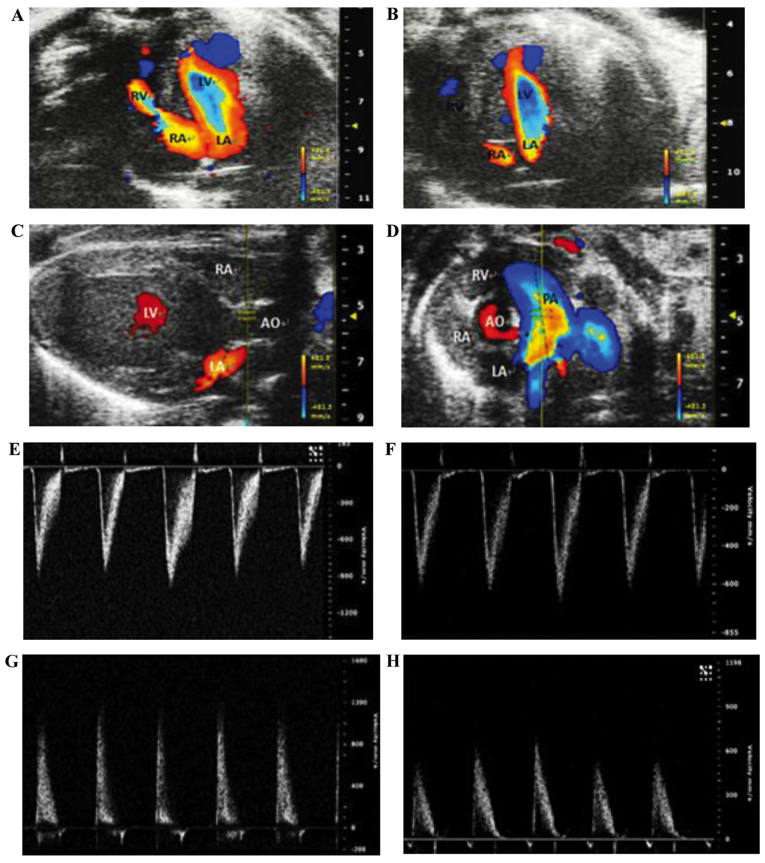
Echocardiography showing an atrial septal defect (ASD) in the heterozygous transgenic mice: (A–D) color Doppler echocardiographic images; and (E–F) pulsed Doppler echocardiographic images. (A) An apical four chamber view of the heterozygous mouse heart, displaying the blood circulation between the atria. (B) An apical four chamber view of the wild-type mouse heart, with no obvious abnormality. (C and D) Images of pulmonary valve peak velocity of the heterozygous transgenic mouse and its wild-type littermate, respectively. (E and F) The pulsed Doppler echocardiographic images across the pulmonary valves of the heterozygous transgenic mouse and its wild-type littermate, respectively. (G and H) The pulsed Doppler echocardiographic images across the aortic valves of the heterozygous transgenic mouse (G) and its wild-type littermate (H). RV, right ventricle; LV, left ventricle; RA, right atrium; LA, left atrium; AO, aorta.

To access the differences in cardiac structure and function between the transgenic mice and their wild-type littermates, 10 transgenic and 6 wild-type mice (8-weeks old) were selected for M-mode echocardiography, two-dimensional echocardiography and color Doppler echocardiography using the Vevo 2100 ultra-high resolution and real-time ultrasonic molecular imaging system. A4C was selected when using color Doppler echocardiography to assess atrial septal integrity. Horizontal sections of the parasternal long axis view of the left ventricle and the parasternal short axis view of the left ventricle (at the aortic valve level) were selected under color Doppler echocardiography and M-mode echocardiography to indicate the aortic and pulmonary artery flow velocities. Among the heterozygous transgenic mice, 8 out of the 10 heterozygous transgenic mice presented with intermittent blood flow occurring between the left and right atria; however, only 1 out of the 6 wild-type littermates presented with the similar symptom (significant difference between groups, P<0.05) (Table III and Fig. 5. The results of color Doppler echocardiography on assessing aortic and pulmonary artery peak velocities and pressure revealed that 6 out of the 10 heterozygous transgenic mice exhibited mild pulmonary stenosis, whereas no abnormality was observed in their wild-type littermates. No statistically significant difference was observed in left ventricular function, left ventricular size and left ventricular wall thickness between the heterozygous transgenic mice and their wild-type littermates (Table IV).

### Expression of GATA4 target genes

**Figure 6:**
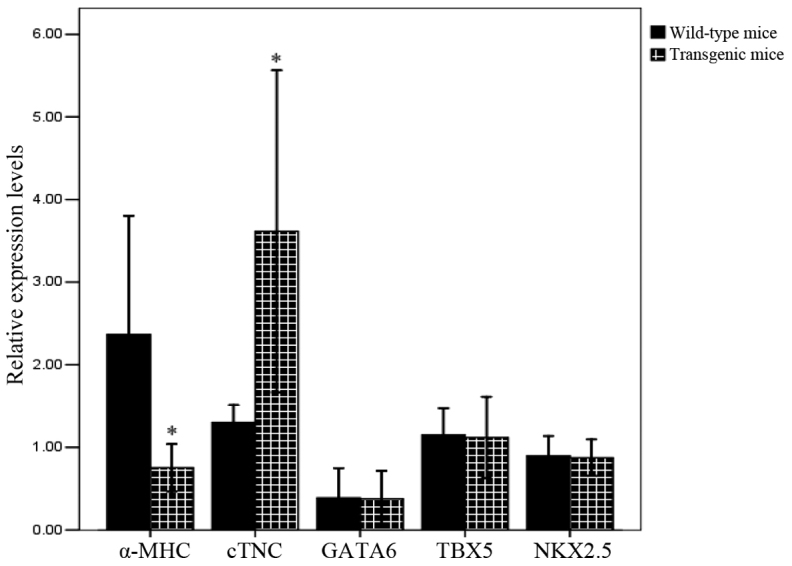
Gene expression in the heart tissue of homozygous transgenic mice. qPCR results demonstrated that the expression of the α-myosin heavy chain (*α-MHC*) gene in the hearts of homozygous transgenic mice was significantly lower than that in the hearts of their wild-type littermates (^*^P<0.05), while the expression of the *cTNC* gene in the hearts of homozygous transgenic mice was significantly higher than that in the hearts of their wild-type littermates (^*^P<0.05). However, no significant difference was observed in the expression of *GATA6*, *TBX5* or *NKX2*.5 between the 2 groups.

To assess the changes in *GATA4* transcript target gene expression in the hearts of teh homozygous transgenic mice, RNA was extracted from the hearts of newborn homozygous mice and qPCR was used to analyze the expression levels of the *α-MHC*, *cTNC*, *GATA6*, *NKX2.5* and *TBX5* genes. The results revealed that expression levels of *α-MHC*, as a *GATA4* downstream target gene, was lower in the hearts of the homozygous transgenic mice than in those of the wild-type mice (P<0.05; Fig. 6), suggesting that the introduction of *GATA4* M310V inhibits the normal expression of this downstream gene. However, the expression level of *cTNC*, as a GATA4 downstream target gene, was higher in the hearts of the homozygous transgenic mice than in those of the wild-type mice (P<0.05), in accordance with the increased protein expression of *GATA4*. There was no change in the expression levels of other *GATA4* downstream genes (*NKX2.5* and *GATA6*) and *GATA4* coactivating genes (*TBX5*). It was thus inferred that the introduction of the *GATA4* M310V exogenous gene suppressed the expression of the *GATA4* downstream gene, *α-MHC*, which may subsequently cause the abnormal development of atrial septum in the hearts of mice.

## Discussion

The findings of the present study are as follows: i) the *GATA4* gene plays an important role in the development of ASD; ii) the *GATA4* downstream gene, *α-MHC*, is associate with *GATA4* function. The altered expression of this gene affects the normal cardiac atrial septal formation during development. This is associated with the incidence of ASD. The conclusions of this study were based on the following: i) in a previous study of ours on *GATA4* mutation screening among patients with CHD revealed that 8 patients with simple ASD from a Chinese family with a history of CHD demonstrated the point mutation of *GATA4* M310V (9). ii) In another previous study of ours, *in vitro* experiments revealed that *GATA4* mutation affected its dose-positive correlation with *α-MHC* activity (16). iii) *GATA4* M310V transgenic mice developed for the purpose of this study revealed ASD phenotypes. iv) *GATA4* downstream gene (*α-MHC*) expression in the hearts of *GATA4* M310V transgenic mice was significantly lower compared with the wild-type littermates (P<0.05). In short, it was here inferred that *GATA4* M310V mutation may result in some functional defects, which suppress the normal expression of *GATA4* downstream genes and affect the normal development of the cardiac septum, resulting in the development of ASD.

ASD, in which blood flows between the left and right atria of the heart, is a very common form of CHD. However, the incidence of ASD and its etiology remain unclear. *GATA4* gene mutations have been demonstrated in many human families and are associated with ASD and pulmonary stenosis (9,11,18). In a previous study by our group, we presented a representative family with familial ASD (9).

The *GATA4* gene is required for normal heart development (2). In addition, the *GATA4* gene is a regulatory factor for heart development and dose-related adjustment (19). Different degrees of *GATA4* gene defect have been found to produce ASD in a mouse model, producing a variety of heart malformations (14). In this study, a *GATA4* M310V transgenic mouse model presenting with ASD phenotypes was developed. Echocardiography and histological examination were used on the cardiac sections to determine the differences in cardiac structure between the heterozygous transgenic mice and their wild-type littermates. The heterozygous transgenic mice carried the same ASD phenotypes as the 8 patients with simple ASD from a previously tested family with CHD. It was here inferred that these specific phenotypes of simple ASD are triggered by *GATA4* gene mutation during heart formation, resulting in partially functional defects in the heart.

*GATA4* is involved in the regulation of the expression of cardiac structural genes, including *α-MHC*, *cTNC* and *ANF* (3,4). It has been found that the conserved *GATA* sites are in the *GATA4* regulatory region of many cardiac promoters and *GATA4* and potently activate their promoters (20). *α-MHC* is the major contractile protein in the heart. Two putative *GATA*-binding sites are in the proximal enhancer of the *α-MHC* gene which suggests that *GATA4* regulates its expression (21). Both *MEF2C* and *GATA4* activate the gene expression of *ANF* and *α-MHC* (22). The *cTNC* gene has been used to examine the molecular mechanisms that regulate cardiac muscle-specific transcription. It has been proven that GATA4 binds specifically to the CEF-1 site of the cTNC enhancer (23). *GATA6*, another member of the GATA family, is also critical to cardiac development and disorders. It has been reported that GATA4 and GATA6 act cooperatively in the heart (24). NKX2.5 is a restrictive transcription factor of the heart and is involved in normal cardiac development and the function of the cardiac conduction system (25–27). *NKX2.5* regulates transcription and often interacts with other transcriptional factors, including *GATA4*, *MEF2C* (25,28,29) and *Hand2* (30). The *TBX5* gene plays a key role in cardiac morphology and the conduction system. Model mice with a missing copy of the T-box transcription factor, *TBX*, have ASD and sporadic ventricular septal defect (VSD) (31).

In a previous *in vitro* study published by our group, we demonstrated that following the introduction of the *GATA4* expression vector using *α-MHC* promoter, the increased concentration of *GATA4* expression vector also enhanced its promoter activity (16) (P<0.05). However, as we demonstrated in the present study, the increased concentration of the mutant *GATA4* expression vector reduced the activity of the *α-MHC* promoter. qPCR revealed that the expression of the *GATA4* downstream target gene, *α-MHC*, in the heart tissues of the homozygous mice was lower than that in the heart tiussues of the wild-type control mice (P<0.05), suggesting that the introduction of *GATA4* M310V inhibits the normal expression of this downstream gene. However, the expression level of *cTNC*, as a *GATA4* downstream target gene, were higher in the hearts of the homozygous transgenic mice than in those of the wild-type mice (P<0.05), in accordance with the increased protein expression of *GATA4*. However, the expression of *GATA6*, *NKX2.5* and *TBX5* was not altered. It was thus inferred that the *GATA4* M310V point mutation may result in the amino acid substitution of methionine to valine in a corresponding position, leading to functional defects in *GATA4*. This change also caused the suppression of downstream gene *α-MHC* expression. The *α-MHC* gene may be associated with the ASD phenotype of *GATA4* M310V transgenic mice.

This study focused on an important familial mutation of the *GATA4* gene found in a clinical study to further establish the findings of basic research. In this study, a *GATA4* M310V transgenic mouse model of ASD phenotypes was developed. Compared with the heart autopsies of embryonically lethal mice and hearts from newborn *GATA4* knockout mice, this transgenic mouse line had a longer survival time and ensured the stable inheritance of the exogenous gene through the transgenic line. This mutant transgenic mouse model may be suitable for research into human CHD. This mouse model may facilitate the study of the genetic and environmental modifications that cause cardiac malformations. It may also help the in-depth understanding of the mechanisms underlying septal defects. This mouse model demonstrated a specific gene mutation which may lead to the functional defects in the animals. Studies on the potential function of *GATA4* genes are warranted to determine the effect and changes associated with *GATA4* mutation.

## Figures and Tables

**Table I:** Primer sequences.

Gene name	Primer sequences
*GATA4* (PCR)	F: CCTTCGACAGCCCGGTCCT R: TGCACAGATAGTGACCCGTCCC
*GATA4* (sequencing)	F: CTGTGCCAACTGCCAGACC R GTGCCCGTAGTGAGATGACAG
*GATA4* (copy no.)	F: GGCCTCTCCTGTGCCAACTGC R CTTCATGTAGAGGCCGCAGGCA
*GCG*	F: AACATTGCCAAACGTCATGATG R: GCCTTCCTCGGCCTTTCA
*GAPDH*	F: CATCACTGCCACCCAGAAGACTG R: ATGCCAGTGAGCTTCCCGTTCAG
*α-MHC*	F: GCTGGAAGATGAGTGCTCAGAG R: CCAGCCATCTCCTCTGTTAGGT
*cTNC*	F: GATGGTTCGGTGCATGAAGGAC R: CTTCCGTAATGGTCTCACCTGTG
*GATA6*	F: ATGCGGTCTCTACAGCAAGATGA R: CGCCATAAGGTAGTGGTTGTGG
*TBX5*	F: CCAAAGACAGGTCTTGCGATTCG R: TTCTCCTCCCTGCCTTGGTGAT
*NKX2.5*	F: GGTCTCAATGCCTATGGCTAC R: GCCAAAGTTCACGAAGTTGCT

α-MHC, α-myosin heavy chain; cTNC, cardiac troponin C; NKX2.5, NK2 homeobox 5; TBX5, T-box transcription factor; GAPDH, glyceraldehyde 3-phosphate dehydrogenase; F, forward; R, reverse.

**Table II:** Results obtained from the heart sections on the incidence of ASD between the transgenic mice and the wild-type mice.

Heart section results	Mouse group	
	*GATA4* M310V heterozygous transgenic mice (n=15)	Wild-type mice (n=20)
ASD	60% (9)^a^	5% (1)
Normal	40% (6)	95% (19)

The Chi-square test was used to analyze the 4-fold table. Fisher’s exact test was used to determine the difference in the incidence of ASD between the transgenic and wild-type mice.

a P<0.05 represents a statistically significant difference between 2 groups. ASD, atrial septal defect.

**Table III:** The echocardiography results about the incidence of ASD in transgenic and the wild-type mice.

Echocardiography	Mouse group	
	*GATA4* M310V heterozygous transgenic mice (n=10)	Wild-type mice (n=6)
ASD	80% (8)^a^	17% (1)
Normal	20% (2)	83% (5)

The Chi-square test was used to analyze the 4-fold table. Fisher’s exact test was used to determine the difference in the incidence of ASD between the transgenic and wild-type mice.

a P<0.05 represents a statistically significant difference between 2 groups. ASD, atrial septal defect.

**Table IV:** Echocardiographic measurements.

Measurement	Wild-type mice (n=6)	Transgenic mice (n=9)
Aortic peak velocity (AV Peak Vel; mm/sec)	788.47±72.71	919.40±195.79
Aortic (AV) peak pressure (mmHg)	2.43±0.53	3.51±1.51
Pulmonary artery peak velocity (PV Peak Vel; mm/sec)	−640.20±32.89^a^	−879.44±213.58
Pulmonary (PV) artery peak pressure (mmHg)	1.66±0.16^a^	3.13±1.63
End-diastolic left ventricular anterior wall thickness (LVAW; day, mm)	0.80±0.07	0.89±0.11
End-systolic left ventricular anterior wall thickness (LVAW; sec, mm)	1.29±0.23	1.45±0.16
End-diastolic left ventricular diameter (LVID; day, mm)	3.30±0.48	3.39±0.28
End-systolic left ventricular diameter (LVID; sec, mm)	2.17±0.66	2.04±0.24
End-diastolic left ventricular posterior wall thickness (LVPW; day, mm)	0.71±0.09	0.80±0.13
End-systolic left ventricular posterior wall thickness (LVPW; sec, mm)	1.16±0.18	1.28±0.17
Ejection fraction (EF, %)	64.17±17.23	71.05±7.28
Fractional shortening (FS, %)	35.29±12.13	39.72±5.79
Left ventricular end-diastolic volume (LV Vol; day, *μ*l)	45.45±14.94	47.49±9.34
Left ventricular end-systolic volume (LV Vol; sec, *μ*l)	17.75±13.17	13.68±4.09
Heart rate (HR, bpm)	491.00±7.75	487.67±6.02

Results are represented as the means ± standard deviation. The Student’s t-test was used to compare the independent samples between 2groups.

a P<0.05 represents a statistically significant difference between 2 groups. Heart rate was maintained at approximately 490 beats/min. Pulmonary artery peak velocity and pressure in the transgenic mice were higher than those in their wild-type littermates (P<0.05).
